# Sedentary Behavior among National Elite Rowers during Off-Training—A Pilot Study

**DOI:** 10.3389/fphys.2017.00655

**Published:** 2017-09-20

**Authors:** Billy Sperlich, Martin Becker, Andreas Hotho, Birgit Wallmann-Sperlich, Mahdi Sareban, Kay Winkert, Jürgen M. Steinacker, Gunnar Treff

**Affiliations:** ^1^Integrative and Experimental Exercise Science, Institute for Sport Sciences, University of Wuerzburg Wuerzburg, Germany; ^2^DMIR Research Group, University of Wuerzburg Wuerzburg, Germany; ^3^L3S Research Center Hanover, Germany; ^4^Institute for Sport Sciences, University of Wuerzburg Wuerzburg, Germany; ^5^Institute of Health Promotion and Clinical Movement Science, German Sport University Cologne Cologne, Germany; ^6^Division of Sports and Rehabilitation Medicine, Ulm University Hospital Ulm, Germany; ^7^Institute of Sports Medicine, Prevention and Rehabilitation, Paracelsus Medical University Salzburg, Austria

**Keywords:** accelerometer, microsoft band 2, multi-sensor, recovery, sedentary behavior, wearable

## Abstract

The aim of this pilot study was to analyze the off-training physical activity (PA) profile in national elite German U23 rowers during 31 days of their preparation period. The hours spent in each PA category (i.e., sedentary: <1.5 metabolic equivalents (MET); light physical activity: 1.5–3 MET; moderate physical activity: 3–6 MET and vigorous intense physical activity: >6 MET) were calculated for every valid day (i.e., >480 min of wear time). The off-training PA during 21 weekdays and 10 weekend days of the final 11-week preparation period was assessed by the wrist-worn multisensory device Microsoft Band II (MSBII). A total of 11 rowers provided valid data (i.e., >480 min/day) for 11.6 week days and 4.8 weekend days during the 31 days observation period. The average sedentary time was 11.63 ± 1.25 h per day during the week and 12.49 ± 1.10 h per day on the weekend, with a tendency to be higher on the weekend compared to weekdays (*p* = 0.06; *d* = 0.73). The average time in light, moderate and vigorous PA during the weekdays was 1.27 ± 1.15, 0.76 ± 0.37, 0.51 ± 0.44 h per day, and 0.67 ± 0.43, 0.59 ± 0.37, 0.53 ± 0.32 h per weekend day. Light physical activity was higher during weekdays compared to the weekend (*p* = 0.04; *d* = 0.69). Based on our pilot study of 11 national elite rowers we conclude that rowers display a considerable sedentary off-training behavior of more than 11.5 h/day.

## Introduction

Elite rowers invest a considerable amount of time for their training averaging >1,000 h per year (Fiskerstrand and Seiler, 2004) i.e., approximately 17% of h per year of waking time. Nevertheless, a great proportion of available time is not spent for training but for recovery including activities of daily living, such as studying, working, traveling etc.

Past investigations focused on analyzing and optimizing the quality of training (Fiskerstrand and Seiler, 2004; Stoggl and Sperlich, 2015), however very little is known about the intensity and volume of physical activity (PA) performed by elite athletes during their off-training time which, as mentioned above, accounts for more than 80% of waking time. This is astonishing as we know that the rate of adaptation (although not exclusively) is an integral of the training stimulus itself (intensity, duration and frequency of stimulus), environmental surrounding, behavior (e.g., nutrition) but also the type of (acute) recovery strategies (Bishop et al., 2008). Largely, this “integrative dose” determines one's individual biological adaptation as well as health.

Surprisingly, to the best of our knowledge only one study so far investigated the PA of elite athletes outside their sport-activity (Weiler et al., 2015) concluding that the elite soccer players were surprisingly sedentary during off-training, especially when compared to non-athletic groups. In this context, recent studies also showed increased prevalence of overweight and obese athletes indicating increased sedentary behavior (Nikolaidis, 2012, 2013). Sedentary behavior as such is defined as any waking behavior characterized by an energy expenditure ≤1.5 metabolic equivalents (MET), while in a sitting, reclining or lying posture (Tremblay et al., 2017). Evidence exists that elevated levels of sedentary behavior in the non-athletic population are associated with various adverse health outcomes, such as cardiovascular disease, diabetes, and all-cause mortality (Chau et al., 2013; de Rezende et al., 2014).

Within the athletic population it is accepted that active when compared to passive (i.e., inactive) recovery (after high-intensity efforts) (Riganas et al., 2015) is likely to impact overall recovery and sport performance (Laursen and Jenkins, 2002; Buchheit et al., 2009). In elite rowers e.g., active compared to passive recovery provides higher rate of lactate removal compared to passive recovery (Riganas et al., 2015) and the active recovery with a more rapid regulation of homeostasis (although not fully understood) may regulate growth and transcription factors (Coffey and Hawley, 2007). In this context, sedentary off-training behavior may negatively affect recovery and in a long-term adaptation to exercise and health.

In summary, analysis of sedentariness in the elite athletic population is rare and only assessed in a team sport setting and not among elite endurance athletes. Potential identification of sedentariness could (i) lead to a change in the view of off-training procedures (e.g., active recovery) and (ii) could stimulate health advice in light of reducing the risk of sedentary-induced all-cause negative health effects due to accustomed in-career sedentary behavior. Therefore, this pilot study aimed to analyse the off-training PA profile in national elite German U23 rowers during 31 days of their preparation period. Based on a previous analysis in football (Weiler et al., 2015) we hypothesized that elite rowers display a considerable sedentary off-training behavior.

## Methods

### Participants

Eleven German U23 rowers, competing at national or international level took part in this investigation (peak oxygen uptake: 66 ± 5 mL·min^−1^·kg^−1^, 20 ± 2 years, body mass: 88.4 ± 9.7 kg, height: 189 ± 7 cm). The inclusion criteria were: (i) age 18–30 years; (ii) male; (iii) squad member of either regional or national level with seamless periods of rowing before study initiation. Exclusion criteria were: (i) medically unfit to perform the study according to previous recommendations (Steinacker et al., 2002). All participants gave their written informed consent to participate in the study which was conducted in accordance with the Declaration of Helsinki. All protocols were pre-approved by the ethical review board of the University of Ulm.

### Assessment of physical activity (PA)

Data collection took place during the final 11-week preparation period (i.e., calendar week 3–14) before the rowers' first competition of the season. Each rower was instructed to wear a wrist-worn multisensory device Microsoft Band II (MSBII), for a period of 1 month (31 days, with 21 weekdays, and 10 weekend days) only removing it for scheduled training sessions and showering. The MSBII incorporates several sensors including a 3-axis accelerometer, gyrometer, optical heart-rate sensor, galvanic skin response sensor, ambient light sensor, ultraviolet light exposure, and skin temperature sensor. The MSB2 stores the data of mean hourly energy expenditure online.

### Preliminary analysis

Beforehand we validated the measurement of energy expenditure of the multi-sensory MSBII with the energy expenditure from indirect calorimetry (Metamax 3B, Cortex, Leipzig, Germany) in nine physical education students. Depending on their level of performance they sat, stood, walked at 3, 4, 5 km·h^−1^ or jogged at 7.2, 9.0, 10.8, and 12.6 km·h^−1^ for 3-min. During each 3-min activity the energy expenditure was measured with the MSBII and a previously validated (Medbo et al., 2002) breath-by-breath metabolic cart (MetaMax 3B, Cortex Biophysik, Leipzig, Germany). In accordance with the manufacturer's instructions both, the gas and flow sensor were calibrated prior to all testing.

**Figure 1:**
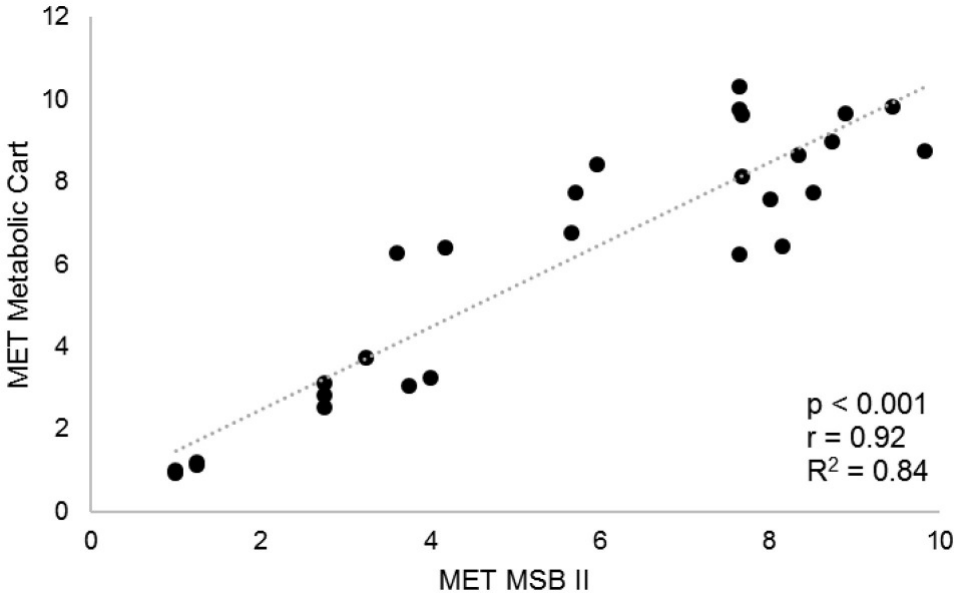
Correlation analysis of energy expenditure measured by MSBII and indirect calorimetry (Cortex Metamax 3B) of nine subjects sitting, standing, walking and jogging.

Over the activity range from 1 to 10 MET (Figure 1), i.e., sitting, standing, walking, and jogging the Pearson correlation coefficient (r) calculation revealed a significant and nearly perfect correlation between the energy expenditure calculated from the multi-sensory MSBII and the energy expenditure from indirect calorimetry (*r* = 0.92; *r*^2^ = 0.84, *p* < 0.001).

The correlation coefficients of this preliminary testing are even higher than previously published correlation coefficients when comparing the energy expenditure assessed by multi-sensor devices and indirect calorimetry in healthy adults [*r* ranging from 0.56 (Fruin and Rankin, 2004) to 0.85 (Dwyer et al., 2009)].

To classify energy expenditure in established PA classifications (Ainsworth et al., 2011; Sedentary Behaviour Research Network, 2012) we normalized the energy expenditure by individual body mass and categorized received mean METs/hour as sedentary activity (<1.5 MET), light (1.5–3 MET), moderate (3–6 MET), and vigorous intense PA (>6 MET). Non-wear time was identified by checking heart rate data, i.e., if no valid heart rate was present for an hour it was deemed that the device must have been removed in that hour. According to the manufacturer, the MSBII automatically tracks the duration of sleep integrating biometric data of heart rate and motion or when the athlete personally activates the sleeping mode. The hours spent in each PA category were calculated for every valid day of data recorded, where a valid day consists of at least 480 min of wear time during waking hours of the non-training period in correspondence with (Atkin et al., 2012). Data classified as time in bed and invalid days (<480 min of wear time) were excluded from the analyses.

### Statistical analysis

The data to calculate the MET values were processed using the Python data analysis toolkit “pandas” (0.18.0) and the scientific computing library “SciPy” (0.17.0) available for the Python programming language (3.5.1). Further analysis was conducted using the Statistica software package for Windows® (version 7.1, StatSoft Inc., Tulsa, OK, USA). That is, a student's paired *t*-test was employed to calculate the differences between weekdays and weekend activities [i.e., sedentary time (<1.5 MET); light PA (1.5–3 MET), moderate PA (3–6 MET); vigorous PA (>6 MET)]. An alpha of *p* < 0.05 was considered as significant. The effect size, Cohen's *d*, (Cohen, 1988) was calculated for all variables, with the thresholds for small, moderate, and large effects set at 0.20, 0.50, and 0.80, respectively (Cohen, 1988). Medium or large effects sizes were considered as tendencies if comparisons based on *p-*values were insignificant.

## Results

A total of 11 rowers provided valid data (i.e., >480 min/day) for 11.6 week days and 4.8 weekend days during the 31-day observation period.

**Figure 2:**
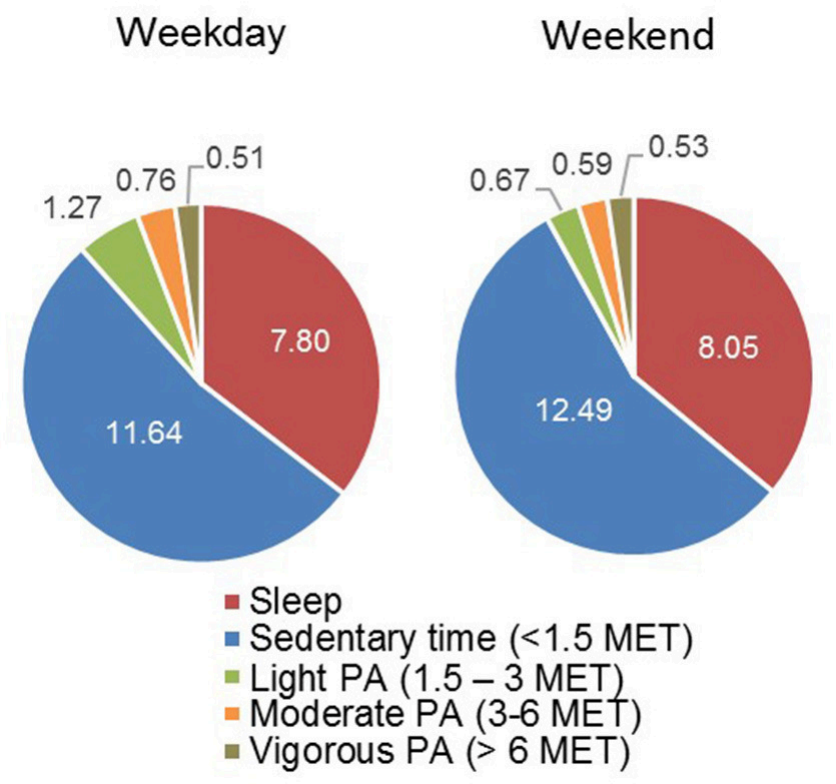
Fraction of mean sedentary time [hours], light, moderate, and vigorous PA as well as sleep of total wear time during off-training periods.

All mean data for sedentary time, light, moderate and vigorous PA as well as sleep are summarized in Table 1 and the corresponding fraction of total wear time during off-training periods are illustrated in Figure 2.

**Table 1:** Summary of daily activity of 11 rowers during their preparation period.

		**Mean ±SD**	**95% CI**	***P*; *d***
Valid days	Weekday	11.55 ± 4.25	8.69–14.40	<0.001^*^
	Weekend	4.81 ± 1.53	3.79–5.85	2.11
Mean wear time per day [h]	Weekday	22.31 ± 1.14	21.54–23.08	0.67
	Weekend	22.05 ± 1.51	21.04–23.07	0.19
Sedentary time (<1.5 MET) in waking hours [h]	Weekday	11.63 ± 1.25	10.80–12.48	0.06
	Weekend	12.49 ± 1.10	11.77–13.21	0.73
Light PA (1.5–3 MET) [h]	Weekday	1.27 ± 1.15	0.49–2.05	0.04
	Weekend	0.67 ± 0.43	0.37–0.96	0.69
Moderate PA (3–6 MET) [h]	Weekday	0.76 ± 0.37	0.51–1.00	0.13
	Weekend	0.59 ± 0.37	0.31–0.87	0.45
Vigorous PA (>6 MET) [h]	Weekday	0.51 ± 0.44	0.20–0.83	0.97
	Weekday	0.53 ± 0.32	0.25–0.80	0.05
Sleep time [h]	Weekday	8.18 ± 1.24	7.35–9.01	0.80
	Weekend	8.07 ± 1.34	7.17–8.97	0.08

PA, Physical activity; d, Cohen's d effect sizes calculated from the Mean ± SD between weekdays and weekend;

* *Indicates differences between weekdays and weekend for P < 0.05*.

The average sedentary time was 11.63 ± 1.25 h per day during the week and 12.49 ± 1.10 h per day on the weekend, with moderate effect sizes indicating sedentary time to be higher on the weekend (*p* = 0.06; *d* = 0.73). The average time per day in light, moderate and vigorous PA during the weekdays was 1.27 ± 1.15, 0.76 ± 0.37, 0.51 ± 0.44 h per day, and 0.67 ± 0.43, 0.59 ± 0.37, 0.53 ± 0.32 h per weekend day. Light activity was higher during weekdays compared to weekend (*p* = 0.04; *d* = 0.69).

## Discussion

In the present study, we aimed to analyze the off-training PA of national elite U23 rowers during their preparation period. To the best of our knowledge this is the first investigation among endurance athletes.

The main findings of this investigation were that national elite U23 rowers when compared to non-athletic population studies (Schuna et al., 2013; Owen et al., 2014) display a larger proportion of time sedentary (<1.5 MET), a lower proportion of light PA, but at the same time display a greater amount of moderate to vigorous PA (>3 MET) in addition to their often vigorous training activity. In their secondary analyses of the NHANES from 2005 to 2006, Schuna and co-workers present a mean sedentary time of 478.9 (2.6) min/day, 200.0 (1.5) min/day in low PA, 141.3 (1.8) min/day in light PA, 87.8 (1.2) in lifestyle PA, and 22.8 (0.7) min/day in moderate-to-vigorous intensity PA (Schuna et al., 2013).

The rowers in the present study spent >11.5 h sedentary i.e., expending a mean metabolic equivalent of <1.5 METs per hour which corresponds to sitting, lying and passive transportation etc. The present data is in line with a previous investigation (Weiler et al., 2015) analyzing professional footballers during an English league season and demonstrating significant sedentary behavior among elite footballers. In the latter study, the footballers spent approximately 8 ± 1 h of waking time sedentary. In the present study, the rowers were about 3.5 h more sedentary (hours per day spend at <1.5 METs) during the weekdays and 4.5 h more sedentary during the weekend. One reason for the calculated sedentariness of our rowers may be attributable to the algorithm (hourly average of activity) of the MSBII neglecting short interruptions of sedentary time with activities of more than 1.5 MET.

However, it is important to note that the sedentariness in our rowers was higher during the weekend compared to weekdays, which has also been confirmed as pattern in other non-athletic populations, such as students (Clemente et al., 2016). Since the rowers were not professional athletes they might not have had enough time (due to work, education, etc.) during the week to perform longer and/or (very) intense sessions. Longer session (and maybe more intense sessions) would lead to fatigue resulting in less off-training activity.

However, the rowers in the present study spent clearly more time (2 min vs. 30 min) at vigorous activity (>6 MET) when compared to elite footballers (Weiler et al., 2015). We can only speculate to why rowers display more vigorous activity during their off-training but maybe this mirrors, at least in part, the typical behavior of rowers preferring more vigorous and exhausting exercise. However, we cannot exclude that some rowers added additional non-scheduled exercise into their free time e.g., a soccer game.

### Active vs. sedentary recovery

To improve recovery, various responses of different modalities have been investigated including macronutrient supplementation (McLellan et al., 2014), massage techniques (Poppendieck et al., 2016), cooling (Poppendieck et al., 2013), self-myofascial release (Beardsley and Skarabot, 2015), neuromuscular electrical stimulation (Babault et al., 2011), active vs. passive recovery (Laursen and Jenkins, 2002; Buchheit et al., 2009; Riganas et al., 2015) (and many more), all of which are performed rather temporarily (minutes to maybe 1 h) and employed promptly after exercise. Short-term active compared to passive recovery in rowers is known to provide a higher rate of lactate removal compared to passive recovery (Riganas et al., 2015) and active recovery with a more rapid regulation of homeostasis (although not fully understood) may regulate growth and transcription factors (Coffey and Hawley, 2007). Similarly, lactic acid clearance measured 20 min after repeated supramaximal leg exercise (i.e., Wingate tests) is significantly greater with active compared to passive recovery and massage in cyclists (Martin et al., 1998). Likewise, young elite futsal players perceive more benefit from immediate postgame (water) exercises compared to dry exercises and seated rest, which is thought to improve their attitude toward playing (Tessitore et al., 2008). In contrast, results indicate that passive and active (i.e., running 5 miles on a flat course on two consecutive days, at an intensity of 65–75% of maximum heart rate) recovery result in similar mean 5-km performance (Bosak et al., 2008). Equally, a single 30-min session of aqua cycling was not able to attenuate the effects on muscular performance, markers of muscle damage, or delayed onset of muscle soreness (DOMS) compared with passive rest (Wahl et al., 2017).

Finally, muscle activation induces blood flow (Sperlich et al., 2013), thereby delivering oxygen and substrates to the muscle and also supports the clearances of metabolites. So, from this perspective, any form of (light) muscle activity during off-training should support circulatory induced recovery.

Based on our experience, active recovery is employed immediately or with time-delay after exercise and for a certain (short) period of time. Since an extremely high variability of “best” recovery scheme exists between different athletes (Bishop et al., 2008) it is astonishing, that no study so far (at least to the best of our knowledge) has investigated the influence of different (long-term) off-training PA profiles in athletes. We acknowledge the fact that certain “sedentary behavior” maybe necessary for elite athletes to properly recover, however the impact of prolonged sedentary behavior during off-training and its impact on athletic recovery, performance or injury risk is unknown. From this perspective, future investigation may aim to answer the question whether the manipulation of off-training PA may be beneficial or harmful for recovery processes and long-term performance development in elite athletes.

### Health risk of sedentariness in athletes?

Although it is well-known that sedentary behavior is related to all-cause mortality (Chau et al., 2013; de Rezende et al., 2014) elite athletes may not be increasingly threatened by this risk (Ekelund et al., 2016). However, Olympic athletes are not immune toward cardio-vascular disorders and might be exposed to unexpected high-risk of cardiovascular abnormalities during sport activity (Pelliccia et al., 2017). Additionally, there is some evidence indicating that elite endurance athletes, when retired, change their body composition more than aerobic characteristics with age (Mujika, 2012). From this perspective, the sedentary behavior of active athletes may not directly be harmful to their health but, especially after retiring from their sporting career, these individuals may be at high risk of sedentary-induced all-cause mortality due to accustomed in-career sedentary behavior.

There is some evidence that interrupting sitting time every 20–30 min by standing up or walking helps to counteract cardio-metabolic disease (Dunstan et al., 2012) and bodies, such as the American College of Sports Medicine address the issue of reducing sedentary behavior (Kravitz and Vella, 2016) repeatedly. The athletic population may not feel addressed, because of their high training related PA. In all cases, athletes should be informed about their current off-training PA profile and the long-term risk associated with sedentary behavior. In this context commercially available wearable sensors (Duking et al., 2016), as long as they fulfill scientific quality criteria (Sperlich and Holmberg, 2017), and do not danger personal data security (Austen, 2015), may be useful in providing feedback (Duking et al., 2017) of daily PA patterns.

### Methodological considerations

Some methodological considerations need acknowledgment: First, we only observed a short period within the season of competitive rowers, i.e., 31 days. Although, this observation period is significantly longer compared to other studies investigating PA patterns (Schuna et al., 2013) we cannot judge whether the PA profile during off- and competition season would be different. Secondly, since our rowers were among the best athletes in Germany we cannot estimate whether the result is also true for recreational, female, youth or older rowers. Thirdly, the data analysis of the MSBII does not allow to record PA densely, i.e., data every second or minute within a 24-h cycle. Consequently, we could not assess the quantity of possible micro bouts of PA, which might have been leveled off through sedentary behavior for the rest of the hour. Also, the position of the wrist-worn device could have an error in the calculation of energy expenditure. Although we instructed all rowers to wear the MSBII always on the same arm we cannot be sure if this was the case all the time.

Also, from a methodological point of view, the number of rowers in the present pilot study was relatively small and more participants would have allowed greater statistical power. However, the 11 rowers were among the best of their age group in Germany and increasing the sample size would have meant to integrate “weaker” rowers thereby confounding the interpretation of the data for the “elite” rowing population. As this study was designed as pilot study, further research is warranted and the present results should be viewed carefully until the data is confirmed in other populations.

### Practical consideration

As mentioned previously (Sperlich and Holmberg, 2017), wearable technology allows to collect as much information as possible to be obtained by continuous 24-h monitoring of various PA and also estimate sleep, and various environmental conditions. As long as scientific quality is ensured (Duking et al., 2016; Sperlich and Holmberg, 2017) and personal data secured, such technology can potentially provide a 24-h feedback (Duking et al., 2017) to the athlete and supporting staff about PA during off-training. Individual feedback to PA may assist to counteract exaggerated sedentariness and could stimulate health advice in light of reducing the risk of sedentary-induced negative health outcomes due to accustomed in-career sedentary behavior.

## Conclusion

Based on our data we conclude that well-trained rowers when compared to other populations display a larger proportion of time sedentary (<1.5 MET) but at the same time display a greater amount of time in moderate to vigorous PA (>3 MET). Future investigation may aim to answer the question whether the manipulation of off-training PA may be beneficial or harmful for recovery processes and long-term performance development and health in elite athletes.

## Author contributions

All designed and approved the methods, analyzed data, and assisted in manuscript writing. BS, MB, BWS, KW, and GT performed data collection.

### Conflict of interest statement

The authors declare that the research was conducted in the absence of any commercial or financial relationships that could be construed as a potential conflict of interest.
